# Quality and Completeness of Myocardial Infarction Recording in Clinical Practice Research Datalink Aurum

**DOI:** 10.2147/CLEP.S319245

**Published:** 2021-08-24

**Authors:** Rebecca Persson, Todd Sponholtz, Catherine Vasilakis-Scaramozza, Katrina Wilcox Hagberg, Tim Williams, Dipak Kotecha, Puja Myles, Susan S Jick

**Affiliations:** 1 Epidemiology, Boston Collaborative Drug Surveillance Program, Lexington, MA, USA; 2 Clinical Practice Research Datalink, Medicines and Healthcare Products Regulatory Agency, London, UK; 3 Institute of Cardiovascular Sciences, University of Birmingham & University Hospitals Birmingham NHS Foundation Trust, Birmingham, UK; 4 Epidemiology, Boston University School of Public Health, Boston, MA, USA

**Keywords:** clinical practice research datalink, CPRD Aurum, data validation, data completeness, data quality

## Abstract

**Background:**

Validation studies of the Clinical Practice Research Datalink (CPRD) Aurum database in the UK are critical for making decisions about its suitability and validity for research purposes.

**Objective:**

To examine data source agreement of myocardial infarction (MI) diagnoses recorded in CPRD Aurum compared with linked Hospital Episode Statistics (HES) data. This comparison provides information on CPRD Aurum data correctness (accuracy, validity) and completeness (presence, missingness).

**Methods:**

Patients with MI diagnoses recorded in either data source were selected from a random sample of 50,000 patients in CPRD Aurum with HES linkage (1997–2017). Correctness was defined as the proportion of MI cases in CPRD Aurum with a concordant MI diagnosis recorded in HES or with strong supporting evidence in either data source. Completeness was defined as the proportion of patients with primary HES-coded MIs with strong supporting evidence that were also present in CPRD Aurum.

**Results:**

There were 1260 patients with MI recorded in the CPRD Aurum sample. The overall correctness of the recorded MI diagnoses was 94%: 986 patients (78%) had concordant diagnoses in HES within 90 days; 123 (10%) were concordant with HES, but with an inconclusive date and another 71 (6%) had strong supporting evidence for being a true MI case. There were 1125 patients with MI recorded in HES primary diagnosis fields with strong supporting evidence in either data source. Of these, 880 (78%) were present in CPRD Aurum, with completeness somewhat higher in more recent years.

**Conclusion:**

MI diagnoses recorded in CPRD Aurum were highly likely to be correct, supporting its use in clinical research studies. Completeness was lower, indicating the need for data linkage for some studies.

## Introduction

Clinical Practice Research Datalink (CPRD) Aurum is a database of United Kingdom (UK) general practice electronic health records sourced from Egton Medical Information Systems (EMIS^®^) patient management software that is newly available to researchers.^1^ Although there are similarities between CPRD Aurum and CPRD GOLD, another UK data source with well-established reliability and quality for conducting medical research,^2–8^ the quality of CPRD Aurum has yet to be fully assessed. Quality assessments of all new databases are necessary to evaluate the suitability of the data as the basis of medical research.

We are conducting several validation assessments of CPRD Aurum using methodologies described by Weiskopf and Weng.^9^ Prior publications have described the quality and completeness of coding for pulmonary embolism, diabetes, hypercholesterolemia and anemia.^10^,^11^ In this paper, we describe the use of data source agreement to evaluate acute myocardial infarction (MI) diagnoses in CPRD Aurum primary care compared with Hospital Episode Statistics (HES), which captures MIs in secondary care. This comparison provides information on “correctness” (ie, accuracy, validity) and “completeness” (ie, presence, missingness) of MI diagnoses recorded in CPRD Aurum. We expect MI diagnoses to appear in both data sources as, in most circumstances, MI requires urgent care in hospital, as well as follow-up care by general practitioners (GPs) and consultants. This study provides an assessment of the validity of MI recording in CPRD Aurum but is also an indicator of the quality of recording of other acute conditions with similar clinical care pathways.

## Methods

### Data Resources

CPRD Aurum is provided by the CPRD, a research service jointly supported by the Medicines and Healthcare products Regulatory Agency and the National Institute for Health Research (NIHR), as part of the UK Department of Health and Social Care. CPRD Aurum is a large, prospectively collected, population-based, anonymized medical record database, which, at the time of data extraction for this study (November 2018), encompassed data on 873 NHS practices and 27.8 million patients. As of April 2021, CPRD Aurum includes 1373 practices and over 39 million patients. Medical records include demographic information, prescription details, clinical events, referrals, hospital admissions, laboratory results, and lifestyle details, such as smoking, alcohol consumption, and height and weight as captured by GPs using the EMIS^®^ patient management software. Apart from emergency medicine, GPs in the UK function as the gatekeepers for all NHS care, including hospital and specialist referrals. Hence, their records are expected to include primary diagnoses leading to hospital referrals. In addition, as secondary care providers are required to send information on patient encounters to the GP, the structured primary care record may also include the primary admitting diagnosis for patients presenting to hospital via other referral routes.^8^ In CPRD Aurum, diagnoses and other non-prescription data are coded using a combination of SNOMED CT (UK edition), Read Version 2, and local EMIS Web^®^ software-specific codes that have been cross-mapped by CPRD to a single diagnostic code (“MedCode”). Prescriptions are coded using the Dictionary of Medicines and Devices (dm+d) codes which are a subset of the SNOMED CT terminology and are assigned a “ProdCode” by CPRD.^1^,^12–14^

HES Admitted Patient Care data, henceforth “HES”, which include information on inpatient hospitalizations in England since 1997 for the purpose of hospital payment, were used as the external reference standard for this validation study.^15^,^16^ All CPRD Aurum practices are linked to HES data. HES data (using ICD-10 codes^17^) contain details of each NHS hospital stay, including dates of admission and discharge, primary diagnosis (ie, reason for hospitalization) and secondary diagnoses made during the hospital stay. Procedures performed during the hospital stay are coded in HES using Office of Population Censuses and Surveys Classification of Surgical Operations and Procedures (OPCS) codes.^18^ Although informative as an external reference standard for the purposes of this study, HES data are not a “gold standard” and may not be more “correct” than CPRD Aurum as they are subject to errors, particularly before the implementation of the Payment by Results (PbR) hospital payment system in 2004–2005.^19^,^20^

### Study Population

We selected a random sample of 50,000 patients in CPRD Aurum during the period 1997–2017 (the period in which both CPRD Aurum and HES records were available) from among practices with a recent HES update in October 2018. To enable comparison, patients were also required to have at least one admission for any reason recorded in HES after the latest of the following: patient’s last EMIS registration date, the patient’s 20th birthday based on year of birth, or the start of HES coverage (April 1, 1997). This 50,000-patient sample was also used for additional validation studies on CPRD Aurum data that describe other data elements and outcomes.^10^,^11^

The start and end of each patient’s active CPRD Aurum electronic record were estimated using the available registration, prescription, and clinical data. Cohort entry was the latest of the patient’s estimated CPRD Aurum start date and April 1, 1997. The end of follow-up was the first of the patient’s estimated CPRD Aurum end date, death date, or the end of available HES data (December 31, 2017). Patients with a diagnosis of MI recorded in CPRD Aurum or HES before cohort entry or patients with a diagnosis of MI recorded on a missing or invalid date were excluded.

### Assessments of Correctness and Completeness of MI Recording

In this assessment, we defined “correctness” of MI diagnoses as the proportion of MI cases recorded in CPRD Aurum that also had a MI diagnosis recorded in HES (“concordance”) or that had strong supporting evidence of MI in either data source to account for coding errors in the HES reference standard. We first found all patients from the study population who had a first-ever diagnosis of MI recorded in CPRD Aurum after cohort entry. The diagnosis and the date of diagnosis of a CPRD Aurum-coded MI were considered concordant with HES if the HES record contained, in any diagnosis field, an ICD-10 diagnosis code for initial or subsequent acute MI (ST elevation MI (STEMI), non-ST elevation MI (NSTEMI) or unspecified acute MI), complications following MI, silent MI, or “old myocardial infarction” recorded within 90 days before or after the CPRD Aurum MI record. The CPRD Aurum MI diagnosis was considered concordant with HES but with an inconclusive date if any of the following were true: 1) the HES diagnosis was recorded at any time during the calendar year for cases in which the CPRD Aurum diagnosis was recorded on January 1 (the date that is assigned by the software when the day and month of an event is not recorded by the GP); 2) a HES diagnosis code for “old myocardial infarction” was recorded on any date more than 90 days after the CPRD Aurum MI diagnosis; 3) the CPRD Aurum MI diagnosis was recorded between 91 and 365 days after the HES MI diagnosis; or 4) other date-related errors were identified (eg, CPRD Aurum MI recorded at next apparent GP visit after the MI was recorded in HES). As HES is not a “gold standard”, CPRD Aurum-coded MIs not concordant with HES were also considered correct if, upon manual review, there was strong evidence of MI based on procedures, referrals, prescriptions or other supporting clinical codes in either data source. CPRD Aurum-coded MIs that were not concordant with HES and did not have strong supporting evidence had a high likelihood of misclassification (ie, it is likely that the patient would be misclassified as an MI case if included in observational research conducted in CPRD Aurum alone).

We then evaluated the completeness of CPRD Aurum data by selecting all patients with an MI diagnosis recorded in a HES primary diagnosis field. (Note: completeness of MI diagnoses recorded in secondary HES diagnosis fields was not assessed in this study, as these comprised <20% of all recorded MIs and were usually secondary to major surgery or other serious conditions such as cancer and thus are not of primary interest for etiologic studies). First, we assessed whether a HES-coded MI was present in CPRD Aurum within 90 days or was present in CPRD Aurum with an incorrect date (same methods described in the correctness section above). Next, to address potential miscoding in HES, we assessed whether the HES-coded MI had strong support in either data source. Completeness was defined as the proportion of patients with HES-coded MIs with strong supporting evidence that were also present in CPRD Aurum. That is, we considered MIs as “missing” in CPRD Aurum only when a HES-coded MI had strong supporting evidence and no corresponding code in CPRD Aurum. These HES-coded, likely MI patients would be missed in research conducted in CPRD Aurum alone.

We calculated estimates of correctness and completeness by sex and age at MI diagnosis. We also stratified all analyses on calendar year to assess the effects of changes to the Quality and Outcomes Framework (QOF) incentives on GP coding and of the implementation of the PbR system on HES coding over time.^20^,^21^

We performed several sensitivity analyses for correctness and completeness estimates, including using shorter (30-day) and longer (ever in record) windows, including only patient diagnoses recorded after 2004 (when the PbR system and data quality QOFs were implemented^20^,^21^), and excluding patients who died or whose records ended within 30 days of the MI diagnosis. Lastly, we explored reasons that the MI diagnosis may have been recorded in one data source and not the other, including issues with data integrity, differential diagnosis between serious CVD diagnoses, and the presence of major comorbidities (eg, cancer).

All CPRD Aurum medical codes and ICD-10 codes for these analyses are found in Supplement 1. All analyses were performed using Stata version 15 (StataCorp, College Station, Texas).

### Ethical Review and Copyright

This study is based in part on data from the Clinical Practice Research Datalink obtained under license from the UK Medicines and Healthcare products Regulatory Agency. The data is provided by patients and collected by the NHS as part of their care and support. The interpretation and conclusions contained in this study are those of the authors alone. This study was approved by the Independent Scientific Advisory Committee (ISAC) for Medicines and Healthcare Products Regulatory Agency (protocol no:18_191A), and the protocol was made available to the journal reviewers upon request. Hospital Episode Statistics (HES) Copyright © (2018), re-used with the permission of The Health & Social Care Information Centre. All rights reserved. Researchers can apply for a limited licence to access CPRD data for public health research, subject to individual research protocols meeting CPRD data governance requirements. More details including data specification, licence fees and applications process are available on the CPRD website (https://www.cprd.com).

## Results

### Study Population

From a random sample of 50,000 patients in CPRD Aurum, we excluded 1170 (2%) with a diagnosis of MI recorded in either CPRD Aurum or HES before cohort entry, as well as 17 (<0.1%) patients with an MI recorded on an unknown or invalid date in CPRD Aurum. Four (<0.1%) additional patients with no recorded birthdate were also excluded. From the final study population of 48,809 patients, there were 1260 (3%) patients with an acute MI diagnosis code in CPRD Aurum and 1434 (3%) patients with an acute MI diagnosis code in HES in any diagnosis field during the follow-up period.

### Correctness of MI Diagnoses Recorded in CPRD Aurum

**Figure 1:**
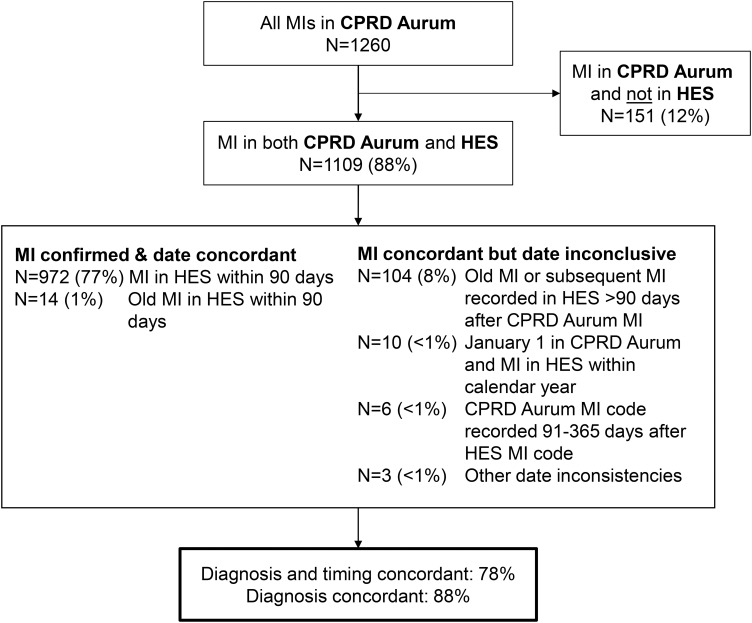
Concordance of MI diagnoses in CPRD Aurum with HES.

The overall correctness of MI diagnoses recorded in CPRD Aurum was high (94%) when assessed using either concordance with diagnoses recorded in HES or strong supporting evidence in either database (Table 1). Of the 1260 patients with an acute MI diagnosis code in CPRD Aurum, 972 (77%) had an MI diagnosis code recorded in HES within 90 days, and 14 (1%) had a code for “old myocardial infarction”. Thus, the diagnosis and the date of 986 (78%) MI diagnoses in CPRD Aurum were concordant with HES. Of these, 806 (64% of all CPRD Aurum-coded MIs) were recorded in both databases within 1 week (510 occurred on the same date). An additional 123 (10%) MI diagnosis codes in CPRD Aurum were concordant with HES, but the date was inconclusive. Overall, 1109 (88%) of first MI diagnosis codes in CPRD Aurum were concordant with HES (Table 1 and Figure 1). The results of most of the sensitivity analyses (30-day HES confirmation window, entire record HES confirmation window, exclusion of patients with MI recorded within 30 days of death or 30 days of the end of record) did not materially change the proportion of cases concordant with HES. However, estimates were somewhat higher when limited to diagnoses recorded in 2005 or later (diagnosis and timing: 85% concordant; diagnosis only: 91% concordant) (Supplement 2).

**Table 1:** Correctness of MI Diagnoses in CPRD Aurum Assessed by Concordance with HES and/or Other Strong Supporting Evidence

Patient Characteristic at Date of CPRD Aurum-Coded MI	N with CPRD Aurum-coded MI	Concordant with HES		Concordant with HES and/or Other Strong Supporting Evidence in CPRD Aurum or HES^‡^, N “Correct” (%)
		Concordant Diagnosis and Timing*, N (%)	Concordant Diagnosis Only^†^, N (%)	
All	1260	986 (78)	1109 (88)	1180 (94)
Sex				
Female	434	324 (75)	371 (85)	401 (92)
Male	826	662 (80)	738 (89)	779 (94)
Age, years				
20–59	302	245 (81)	281 (93)	290 (94)
60–79	622	480 (77)	548 (88)	584 (94)
80+	336	261 (78)	280 (83)	306 (91)
Calendar year				
1997–1999	127	80 (63)	109 (86)	120 (94)
2000–2004	348	239 (69)	288 (83)	315 (91)
2005–2009	278	217 (78)	238 (86)	256 (92)
2010–2014	310	269 (87)	288 (93)	300 (97)
2015–2018	197	181 (92)	186 (94)	189 (96)

**Notes**: *Number of patients with a HES MI diagnosis code or old MI diagnosis within 90 days of first CPRD Aurum-coded MI. ^†^Number of patients with a HES MI diagnosis code or old MI diagnosis confirming the patient’s CPRD Aurum-coded MI, regardless of timing (see methods). ^‡^CPRD Aurum-coded MI concordant with HES and/or presence of strong evidence of MI based on procedures, referrals, prescriptions or other supporting clinical codes in either data source. See Figure 2.

**Figure 2:**
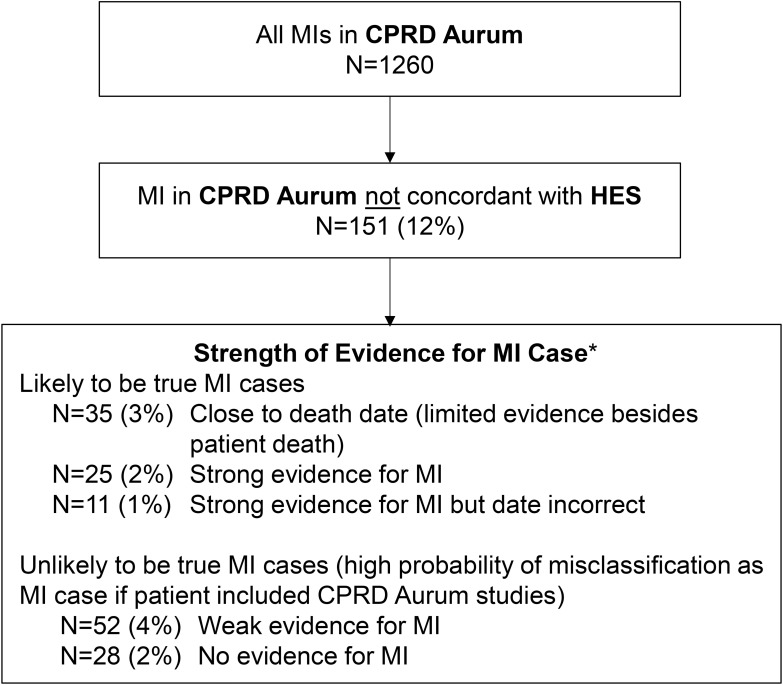
Strength of supporting evidence for CPRD Aurum-coded MIs not concordant with HES. *Based on number, type and timing of diagnosis, procedure, referral, and treatment codes recorded in CPRD Aurum and/or HES around the date of the CPRD Aurum-coded MI.

There were 151 patients with an MI diagnosis code in CPRD Aurum with no concordant diagnosis in HES. Of these, 35 died within 30 days and, thus, there was little follow-up available to assess whether these MI cases were supported by other evidence. For those patients who did not die, we assessed the likelihood that the MI was correctly coded based on all other available diagnosis, procedure, referral, and treatment data in CPRD Aurum and HES. There were 25 patients whose records provided strong evidence for the MI diagnosis (eg, specific MI code such as a STEMI, multiple MI diagnosis codes) and an additional 11 patients who were also likely true MI cases but with unknown or incorrect timing (often recorded on January 1 in CPRD Aurum). Of the remaining 80 patients (6% of all MIs recorded in CPRD Aurum), there were 52 (4% of MIs recorded in CPRD Aurum) where the diagnosis had only weak evidence for MI in HES and CPRD Aurum (eg, ischemic heart disease diagnosis with coronary and cardiac imaging and/or cardiology referrals) and 28 (2%) where the MI diagnosis had no relevant evidence in either CPRD Aurum or HES (Figure 2). Supplement 3 provides additional description of why a CPRD Aurum-coded MI may not have been found in HES.

Thus, the correctness of MI diagnoses recorded in CPRD Aurum was high (94%) with only 6% of CPRD Aurum-coded MIs unlikely to be true MI cases (Table 1). Correctness was high across sexes and ages. Although the proportion of CPRD Aurum-coded MIs concordant with HES (using both diagnosis and date criteria) was low in the years before 2005 (<70%), correctness was high across all calendar years as most early diagnoses had sufficient supporting evidence to be considered correct (Table 1).

### Completeness of MI Diagnosis Recording in CPRD Aurum

**Figure 3:**
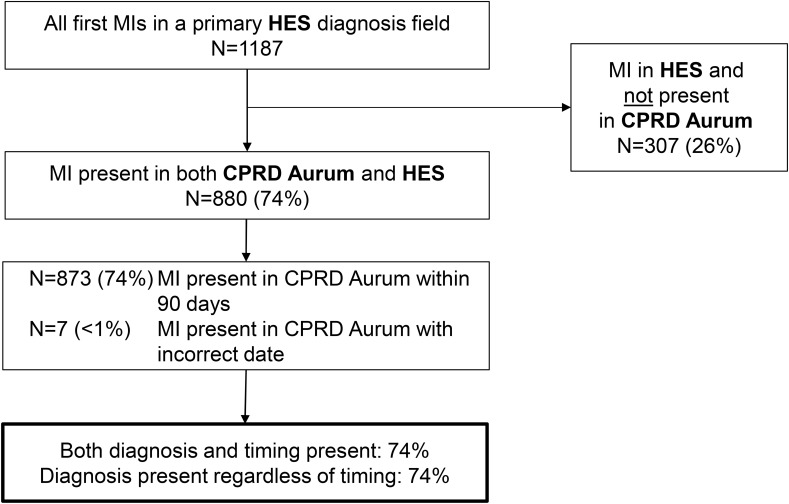
Number of primary HES-coded MI diagnoses present in CPRD Aurum.

The overall completeness estimate for MI diagnosis recording in CPRD Aurum was 78%. There were 1187 patients with a first acute MI diagnosis code in a primary HES diagnosis field during the follow-up period. Of these, 880 (74%) had an MI diagnosis in CPRD Aurum, nearly all recorded within 90 days. Of these, 701 (59% of all HES-coded MIs) were recorded in CPRD Aurum within one week (440 on the same date) (Figure 3).

**Figure 4:**
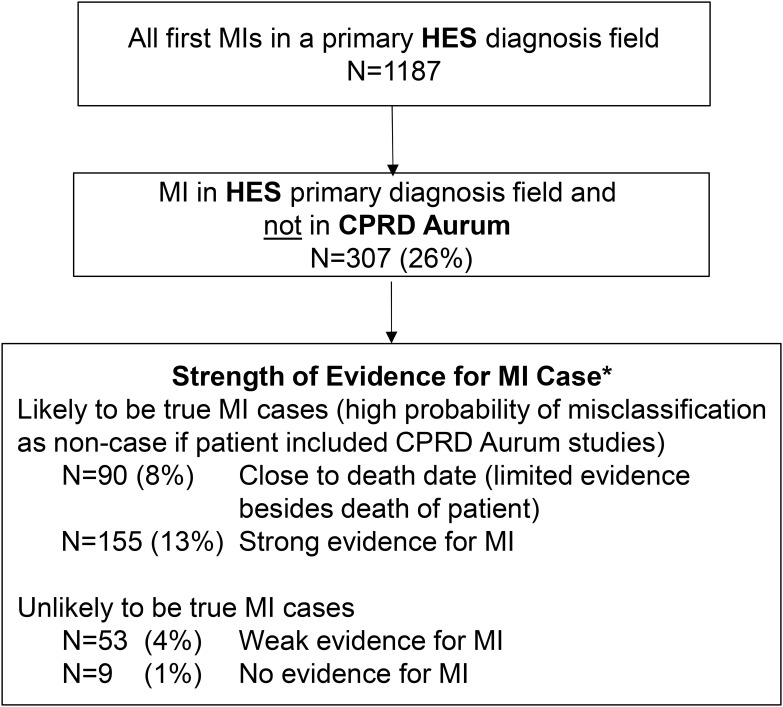
Strength of supporting evidence for HES-coded MIs not recorded in CPRD Aurum. *Based on number, type and timing of diagnosis, procedure, referral and treatment codes recorded in CPRD Aurum and HES around the date of the HES-coded MI.

Among the remaining 307 patients with MIs recorded in HES and not in CPRD Aurum, 245 had either strong evidence in one or both data sources that the MI occurred (eg, specific MI codes such STEMI or NSTEMI in HES, MI code paired with a stent, bypass or angioplasty procedure in HES, or cardiac rehabilitation recorded in CPRD Aurum following the HES MI) or died within 30 days (Figure 4). There was only weak evidence that the MI occurred (eg, unspecified MI with cardiac or coronary imaging codes in HES paired with “attachment” codes in CPRD Aurum) in 53 of the remaining MI cases not recorded in CPRD Aurum (4% of all primary HES-coded MIs). Finally, 9 (1% of all primary HES-coded MIs) had no supporting evidence of MI in either HES or CPRD Aurum (Figure 4). Supplement 4 provides additional description of why a HES-coded MI may not have been found in CPRD Aurum.

Thus, there were 1125 primary HES-coded MIs with strong supporting evidence (or patient death) of which 880 were found in CPRD Aurum, resulting in a completeness estimate of 78%. Completeness was higher for males and for younger patients and increased over calendar time (Table 2). Completeness estimates did not materially change when we conducted sensitivity analyses using shorter or longer time windows to identify codes in CPRD Aurum or when limited to diagnoses recorded in HES in 2005 or later. Completeness was higher (~85%) when analyses excluded patients with MI recorded in HES within 30 days of death or the end of their record (Supplement 5).

**Table 2:** Completeness of MI Recording in CPRD Aurum Estimated as the Number of Primary HES-Coded MIs Also Found in the CPRD Aurum Patient Records

Patient Characteristic at Date of Primary HES-Coded MI	All Primary HES-Coded MIs		Primary HES-Coded MI and Strong Supporting Evidence in Either Data Source †	
	N Total	N (%) Present in CPRD Aurum *	N Total	N (%) Present in CPRD Aurum *
All	1187	880 (74)	1125	880 (78)
Sex				
Female	404	281 (70)	378	281 (74)
Male	783	599 (77)	747	599 (80)
Age, years				
20 – 59	275	235 (85)	267	235 (88)
60 – 79	573	426 (74)	546	426 (78)
80+	339	219 (65)	312	219 (70)
Calendar year				
1997 – 1999	119	82 (69)	110	82 (75)
2000 – 2004	290	217 (75)	275	217 (79)
2005 – 2009	272	187 (69)	253	187 (74)
2010 – 2014	289	225 (78)	272	225 (83)
2015 – 2018	217	169 (78)	215	169 (79)

**Notes**: *Number (%) of patients with a primary HES-coded MI also recorded in CPRD Aurum. ^†^This is a subset of all primary HES-coded MIs who also had strong evidence of MI based on procedures, referrals, prescriptions or other supporting clinical codes in either data source. See Figure 4.

## Discussion

This study demonstrates that MI diagnosis recording in CPRD Aurum is sufficient for most observational research. Throughout the study period (1997–2018), >90% of MI diagnoses recorded in CPRD Aurum were concordant with HES or had strong supporting evidence for the diagnosis. The completeness of CPRD Aurum was somewhat lower, with 78% of primary HES-coded MIs with supporting evidence also present in CPRD Aurum. Completeness varied over the study period (74–83%) and was highest in 2010 and later. Our findings support the use of CPRD Aurum for clinical research on MI and similar acute conditions.

We chose to examine the recording of MI diagnoses as part of our assessment of CPRD Aurum as it is a serious acute condition that requires medical attention where the patient care pathway spans both primary (GP) and secondary (hospital) healthcare settings. For this reason, we would expect that any patient who had a true non-fatal MI would have a diagnosis recorded in CPRD Aurum and in HES data. However, it is possible that some patients with a true MI received care entirely in outpatient setting (particularly older, multimorbid patients). Private hospital care is also possible but exceedingly rare in the UK system. It is also possible that HES data were incorrect, as coding is often performed by non-clinical hospital administration staff. Discharge letters (written by physicians to GPs) often clarify in text the conditions surrounding various diagnosis codes, which may have influenced whether GP staff coded for this condition (particularly where MI was not primarily an issue with the coronary arteries but due to a secondary condition such as sepsis). Thus, where a MI diagnosis was not recorded in both data sources, we assessed the likelihood that the MI was a true case based on all available diagnosis, procedure, referral, and treatment data. Ultimately, the number of patients with “incorrect” MI diagnosis codes in CPRD Aurum (proportion of patients potentially misclassified as cases in epidemiological studies of MI) was low (<10%). However, the proportion of primary MI cases missed in CPRD Aurum was as high as 22%. These cases would be missed in epidemiological studies of MI using CPRD Aurum alone. It is important to note that, given the presence of free text and attachments, GPs likely know about the patient’s MI status; however, the diagnosis is not always recorded in a way that makes it available to researchers.

The concordance of the two data sources increased over calendar time, likely due, at least in part, to a more robust implementation of electronic data quality in the UK, both at primary care level (QOFs) and secondary care level (PbR system).^20^,^21^

Although correctness of recorded MI diagnoses was relatively high, users of CPRD Aurum data should be aware that as many as 10% of MI diagnoses present in CPRD Aurum may be recorded on an imprecise date (particularly if recorded on January 1) or may represent a pre-existing condition (particularly if recorded on the same day as codes related to a regular check-up appointment such as height and weight). Furthermore, the most common reason that primary HES-coded MIs were missing from CPRD Aurum was patient death.

HES is not a perfect reference standard since the majority of coders are non-clinical staff and due to non-specific coding of some hospital events. In this study, we required all CPRD Aurum patients selected for this random sample to have at least one admission for any reason in HES within their follow-up. This was necessary to have two data sources to compare. Thus, the measures presented here may be under- or overestimates. However, the goal of this study was to assess the quality of diagnosis recording in the CPRD Aurum data source, rather than to estimate unbiased measures of sensitivity and specificity. Formal validation studies are still needed to assess the validity of each outcome under investigation. Furthermore, this study does not address information bias that may arise due to differences between a “correct” diagnosis code and the true clinical condition of a patient.

The findings of this study are consistent with two prior assessments conducted in this same data sample:^10^,^11^ diagnoses recorded in CPRD Aurum are of relatively high correctness for use in medical research; however, completeness may not be sufficient for all research questions. When using CPRD Aurum data, researchers should consider study design choices or the addition of linked data. For example, one should consider using a combination of CPRD Aurum and linked data, such as HES and death registry data, to improve capture of study events.^22^,^23^
